# Hope and despair in cancer and other illnesses: a clinical framework for adaptive hope across the illness trajectory

**DOI:** 10.1016/j.esmoop.2026.108340

**Published:** 2026-07-24

**Authors:** N.I. Cherny, H.M. Chochinov, L. Emanuel, C. Lethborg, N. Coyle, B.W. Corn

**Affiliations:** 1 Norman Levan Center for Humanistic Medicine, Helmsley Cancer Center, Shaare Zedek Medical Center, Jerusalem, Israel; 2 Hebrew University, Jerusalem, Israel; 3 CancerCare Manitoba Research Institute, University of Manitoba, Manitoba, Canada; 4 Feinberg School of Medicine, Northwestern University, Chicago, USA; 5 Department of Medicine, Faculty of Medicine, Dentistry and Health Sciences, University of Melbourne, Melbourne, Australia; 6 Supportive Care Service, Department of Medicine, Memorial Sloan Kettering Cancer Center, New York, USA; 7 Department of Oncology, St. Vincent’s Hospital, Melbourne, Australia

**Keywords:** advanced cancer, communication, distress, hope, miracles, serious illness

## Abstract

Hope and despair are central and often coexisting dimensions of the experience of cancer and other serious illnesses. Hope profoundly shapes how patients understand prognosis, engage with treatment, and make decisions across the illness trajectory. When poorly managed, hope may contribute to prognostic misunderstanding, burdensome overtreatment, erosion of trust, and clinician distress. This narrative review synthesizes empirical and conceptual literature on hope and despair in oncology and serious illness, examining their determinants, clinical manifestations, and implications for communication and decision making. Hope is presented as a dynamic, multidimensional construct that evolves over time. Although initially oriented toward cure or remission, hope often recalibrates toward achievable goals such as symptom control, maintenance of function, relational connection, meaning, dignity, reconciliation, and spiritual peace. Hope and despair are shaped by physical symptoms, psychological state, social context, cultural frameworks, spirituality, and existential concerns. Hope for exceptional outcomes or miracles is common and may coexist with realistic planning and adaptive coping. However, when hope becomes rigid or reality-incongruent, it may impair informed decision making and delay emotional and practical preparation for decline and death. Effective clinical care requires the capacity to sustain adaptive, reality-congruent hope through honest, compassionate, and relationally grounded communication. The review examines psychospiritual, relational, and interdisciplinary approaches that support adaptive hope, including empathic communication, symptom management, meaning-centered interventions, dignity-conserving care, spiritual support, and structured hope-enhancing strategies. The role of clinicians’ own reflective and emotional capacities in sustaining hope-supportive care is also explored. Fostering adaptive hope is presented as a core clinical competency in oncology and serious illness care. Integrating communication training, interdisciplinary collaboration, and existentially informed care may improve patient-centered decision making, reduce distress, and enhance the quality of care across the illness trajectory.

## Highlights

• Hope and despair are dynamic, coexisting states across chronic and life-limiting illness trajectories.
• Hope extends beyond cure, evolving toward meaning, connection, and achievable goals.
• Poorly managed hope contributes to overtreatment, mistrust, and clinician moral distress.
• Fostering adaptive hope is a core clinical competency in oncology.
• Compassionate, honest communication enables adaptive hope to evolve without deception.

## Introduction

For clinicians, and especially oncologists, who routinely care for patients confronting life-limiting disease, hope is not a peripheral concern but a core clinical domain.^1^^,^^2^ The capacity to recognize, interpret, and respond to the evolving issues of hope and despair is a professional competency that influences patient outcomes, the quality of decision making, and the integrity of the therapeutic relationship.

Patients living with acute, chronic, or life-limiting illness—including advanced malignancies, progressive organ failure, neurodegenerative conditions, and complex multimorbidity—face not only biological deterioration but also existential threat. Across these trajectories, individuals may experience loss of function, escalating symptom burden, dependence, and heightened awareness of mortality. Within this context, hope serves as a critical psychological and existential resource, while despair represents an equally intelligible response to perceived loss, threat, and uncertainty.

Importantly, despair does not arise solely from proximity to death. It may emerge from uncontrolled symptoms, loss of autonomy, disruption of identity and social roles, moral distress, or a sense of abandonment by clinicians. Conversely, hope is rarely confined to expectations of a cure. Patients may hope for symptom relief, meaningful time, reconciliation, dignity, spiritual peace, legacy, or simply the preservation of a tolerable day. As illness evolves, so too does the object of hope, often shifting in ways that reflect an adaptive recalibration to biological reality.

Dealing with these issues requires a nuanced capacity to sustain and support hope without recourse to deception. Failure to do so carries substantial risks. When clinicians lack the skills to engage hope constructively, they often engage in implicit or explicit deception by overstating the benefits of treatment, avoiding prognostic clarity, or colluding with unrealistic expectations. Particularly, near the end of life, this can contribute to overtreatment, exposing patients to burdensome and often harmful interventions that are misaligned with their values and are unlikely to confer meaningful benefit.^3^ These untoward outcomes can contribute to moral distress, imposter syndrome, and ultimately burnout,^4^ as clinicians struggle to reconcile their actions with their ethical commitments. Furthermore, when patients or families perceive inconsistency, evasion, or false reassurance, this can undermine trust.

To be better equipped to support adaptive forms of hope (hope that is honest, flexible, and aligned with patients’ evolving goals and realities), clinicians, and especially oncology clinicians, require deep familiarity with determinants and expressions of hope and despair. Despite this, hope is rarely addressed in medical training, and many physicians struggle with the management of terminally-ill patients who maintain hope for cure.^5^ This review addresses the following central question: How can clinicians understand, interpret, and support adaptive forms of hope while recognizing and addressing despair across the trajectory of serious illness, particularly advanced cancer?

## Methodological approach

Given the multidimensional nature of hope—encompassing psychological, existential, relational, cultural, and spiritual domains—and the inclusion of both empirical and conceptual literature, a narrative review methodology was selected. This approach was considered most appropriate for integrating diverse theoretical frameworks, clinical observations, ethical analyses, and intervention studies into a clinically applicable framework.

Literature was identified through iterative searches of PubMed, PsycINFO, and Google Scholar using combinations of the terms ‘hope’, ‘despair’, ‘advanced cancer’, ‘palliative care’, ‘existential distress’, ‘demoralization’, ‘meaning-centered therapy’, ‘communication’, and ‘serious illness’.

Priority was given to empirical studies, conceptual analyses, seminal theoretical works, communication frameworks, and intervention studies relevant to oncology and palliative care. The selection prioritized literature that had a substantial influence on clinical practice, psycho-oncology, palliative care, communication science, and existential care frameworks relevant to serious illness. Both quantitative and qualitative literature were included. Since the review was not intended to be exhaustive but rather interpretive and clinically relevant, literature was selected based on conceptual relevance, clinical applicability, and contribution to understanding adaptive and maladaptive forms of hope across illness trajectories. Key historical and foundational works were incorporated whenever conceptually important.

The authors’ perspectives are informed by longstanding clinical and scholarly engagement in oncology, palliative care, psycho-oncology, existential psychotherapy, communication research, and ethics. This includes extensive clinical experience working directly with patients and families facing serious illness. We recognize clinical experience not only as context but also as a valuable source of knowledge that informs sensitivity to the relational, cultural, and context-dependent nature of hope, meaning, and communication in practice. The interpretive synthesis presented reflects this integration of clinical and scholarly perspectives, with a deliberate emphasis on practical relevance for clinicians.

## Despair in illness

### Defining despair

Despair in illness rarely arises from a single cause; rather, it reflects the cumulative impact of physical suffering, psychological distress, social disconnection, cultural meaning, and existential threat.^6^^,^^7^ Despair is a profound sense of hopelessness, helplessness, and loss of meaning, often accompanied by the conviction that no positive future remains imaginable.^8^ It emerges when suffering feels uncontrollable, when decline appears inevitable, or when one’s sense of self is threatened by progressive loss of function, independence, or dignity. It can be intensified by anticipatory grief, whether related to death, disability, or erosion of identity.^8^^,^^9^

Despair is not confined to those who are imminently dying. Patients living for years with advanced organ failure, neurodegenerative disorders, complex multimorbidity, or refractory chronic illness may experience recurring or persistent despair as they confront accumulating losses, uncertain trajectory, and shrinking horizons of possibility. Furthermore, the temporal ambiguity of chronic illness, neither clearly terminal nor clearly stable, may deepen this distress. Even in acute illness, patients may experience a sudden disruption of their anticipated life course, such that the assumptions about the future that previously structured their lives no longer hold.

### Clinical manifestations of despair

Clinically, despair can present in diverse and overlapping ways across illness trajectories. Patients may experience persistent emotional distress, including sadness, anxiety, or apprehension about the future.^6^ Many report a loss of purpose or meaning, describing life as no longer worthwhile or perceiving previously valued goals as unattainable.^10^^,^^11^ Others articulate a sense of being trapped—either in a body that no longer functions as desired or in a life increasingly defined by medical dependency.

Social withdrawal is frequent, often arising from feelings of burden, misunderstanding, or alienation. In chronic or progressive illness, this withdrawal may develop gradually as functional limitations restrict engagement in work, family roles, or community activities. For some, despair acquires a spiritual or existential dimension, manifesting as doubt, anger, or questioning the fairness or purpose of suffering.^12^

Despair is not always verbally expressed. It may appear through disengagement, refusal of care, irritability, diminished adherence, or quiet resignation—behaviors that can be misinterpreted as acceptance, stoicism, or realism. Particularly, in chronic illness, the normalization of decline can obscure profound internal suffering, leaving emotional distress unrecognized by caregivers and health care providers.

#### Vignette 1: unspoken despair

Luke, a 69-year-old man with advanced lung disease, rarely articulated sadness or fear. He attended appointments, complied with treatment, and told clinicians he was ‘fine’. Over time, however, he became increasingly withdrawn, declined visits from family, and refused symptom-relieving interventions, stating he ‘didn’t want to be a burden’. His quiet disengagement was initially interpreted as acceptance. Careful exploration revealed profound despair rooted not only in breathlessness and fatigue, but also in the erosion of identity as an independent husband, worker, and father. His suffering was less about imminent death than about progressive diminishment of self.

### Contributors to despair

#### Physical contributors

Uncontrolled symptoms—including pain, breathlessness, fatigue, nausea, insomnia, and cognitive decline—are powerful drivers of despair.^13^^,^^14^ Even the anticipation of uncontrolled symptoms, particularly pain or suffocation, can generate profound anxiety.^15^ In chronic disease, recurrent exacerbations and stepwise decline may repeatedly contribute to despair. This physical deterioration, even when it is slow, can overwhelm coping capacity.^16^

#### Psychological contributors

Anxiety and depression are particularly common in advanced and chronic illness and can overshadow hope.^17^ Cognitive distortions—catastrophic thinking, all-or-nothing reasoning, learned helplessness—may amplify despair.^18^ For patients with fluctuating trajectories, uncertainty itself becomes a psychological burden.

Fear of the unknown—concerns about progressive disability, cognitive loss, dependency, institutionalization, or the dying process—often looms large.^19^ Paradoxically, rigid or unrealistic optimism—whether self-generated or socially imposed—can exacerbate despair when reality intrudes.^20^

#### Social contributors

Social isolation is a potent source of despair. Functional impairment, fatigue, and stigma may progressively narrow social worlds.^21^ Loss of meaningful professional, parental, and communal roles can be profoundly destabilizing.

Unresolved interpersonal conflict, guilt, anger, shame, or regret may further burden patients and compound distress.^22^^,^^23^ In chronic illness, prolonged dependency may strain relationships, heightening feelings of burdensomeness.^24^ When families avoid open conversations about prognosis or shield patients from difficult truths, patients may experience emotional loneliness and relational inauthenticity.^25^^,^^26^

#### Cultural contributors

Cultural context profoundly shapes how illness, disability, and dying are understood and expressed. Stigma, norms valorizing stoicism, collective trauma, and culturally embedded expectations regarding prognostic disclosure influence whether distress is voiced openly or concealed.^27^^,^^28^ Norms encouraging emotional restraint may discourage help-seeking behavior.^29^^,^^30^ Conflicts between culturally grounded values and prevailing medical practices—particularly regarding prognostic disclosure, autonomy, and decision-making authority—may intensify distress when patients and families feel caught between competing frameworks.^31^

#### Existential contributors

Existential fear is a central component of despair.^32^, ^33^, ^34^ Fear of death may coexist with a fear of prolonged decline, cognitive dissolution, or loss of relational recognition.^35^ The erosion of identity—no longer fully inhabiting roles as parent, professional, partner, or independent adult—threatens coherence of self.^36^^,^^37^ Patients may feel reduced to diagnoses, laboratory values, and functional scores rather than recognized as whole persons.

### Is despair inevitable?

Although despair and existential distress are common in illness, they are not inevitable, even for chronic and life-limiting illness. Their severity, duration, and impact vary widely among individuals.^10^^,^^32^ Patients frequently oscillate between despair and hope, even within the same day.^38^ Recognizing this dynamic variability helps clinicians avoid pathologizing despair while remaining attentive to opportunities for relief, reconnection, and restoration of meaning.

Comprehensive psychosocial and meaning-centered interventions addressing physical, emotional, relational, and spiritual domains reduce despair and demoralization in advanced disease.^39^, ^40^, ^41^ Such approaches are equally relevant across chronic illness trajectories.

#### Vignette 2: oscillation between despair and hope

A 62-year-old woman living with metastatic cancer described mornings overshadowed by the certainty of decline and evenings spent planning visits with her grandchildren. On some days, she spoke of being ‘tired of fighting’; on others, she expressed gratitude for small pleasures and moments of connection. These fluctuations did not reflect denial or inconsistency but, rather, the natural oscillation between despair and hope characteristic of chronic and life-limiting illness trajectories.

## Hope in illness

### Defining hope

Hope has been conceptualized across psychological, philosophical, existential, and theological traditions. Aristotelian virtue traditions understand hope in relation to human flourishing and moral orientation toward the good,^42^ while many religious traditions frame hope in relation to transcendence, redemption, or continuity beyond death.^43^ The present review adopts a clinically oriented multidimensional framework integrating psychological, relational, existential, and spiritual dimensions of hope as they arise in serious illness.

Hope in the context of serious, chronic, and life-limiting illness is frequently misconstrued as synonymous with belief in cure or prolonged survival. It is, however, a far more complex and multidimensional phenomenon. Hope may be understood as the sustained belief in the possibility of a positive outcome, even in the face of uncertainty or adversity. As such, it functions as an emotional bridge between an unsatisfying present and a possible better future.

In chronic and life-limiting illness, hope is not reducible to a single desired outcome; rather, it functions as a dynamic, adaptive process. As circumstances shift, whether gradually over years or abruptly with clinical deterioration, hope evolves, enabling patients to remain engaged with life, relationships, identity, and purpose even as physical decline progresses.

The most widely studied model of hope is Snyder’s Hope Theory,^44^ which conceptualizes hope as comprising three interrelated elements as follows:

• Goals—personally meaningful aims that provide direction. These may include recovery, cure, prolonged stability, time with family, attending a milestone event, reconciliation, symptom relief, maintaining independence, spiritual peace, or living life ‘to the last drop’. In chronic illness, goals may be modest and immediate such as walking to the mailbox, remaining at home, and preserving cognitive clarity. Goals are not fixed; hope allows them to shift as circumstances change.
• Pathways—the perceived ability to generate routes toward those goals. When one path becomes blocked by disease progression or functional loss, hopeful thinking seeks alternatives.
• Agency—the motivational energy that sustains movement toward valued aims, reflecting determination and engagement rather than naïve confidence.

This model underscores the fluidity of hope. When cure becomes unattainable, hope may reorient toward comfort, connection, adaptation, or meaning. In chronic illness, hope often recalibrates repeatedly over time in response to exacerbations, remissions, and accumulating losses. Hope persists even as its objects change. While Snyder’s Hope Theory has been influential because of its operational clarity and emphasis on agency and pathways, broader multidimensional formulations may be particularly relevant in palliative-care contexts.

Dufault and Martocchio^45^ conceptualize hope as a multidimensional, dynamic life force that serves as an active, future-oriented source of energy essential for coping with severe illness, existential distress, and the end of life. The model distinguishes generalized hope, which reflects a broad sense of openness, inner resilience, and trust in the future, and particularized hope, which is directed toward specific desired outcomes such as cure, symptom relief, or meaningful life events. In this model, hope is further understood through the following six dimensions:

1. An affective dimension, involving feelings of confidence and positivity;
2. A cognitive dimension, encompassing realistic appraisal, goal setting, and interpretation of circumstances;
3. A behavioral dimension, reflected in actions and perseverance toward desired aims;
4. An affiliative dimension, highlighting the sustaining role of emotionally meaningful relationships and caregiving connections;
5. A temporal dimension, through which hope integrates meaning from the past, endurance in the present, and anticipation of the future;
6. A contextual dimension, recognizing that hope is shaped by the individual’s environment, life situation, and surrounding circumstances.

Some frameworks in positive psychology and virtue ethics conceptualize hope as a character strength or virtue. For example, the Values in Action Classification of Strengths and Virtues,^46^ developed within positive psychology, situates hope among the core virtues associated with transcendence, meaning, and positive future orientation. Although virtue-based traditions conceptualize hope as a relatively stable moral orientation or character strength, the present review primarily examines hope phenomenologically and clinically as it emerges and evolves within serious illness.

Importantly, hope differs from optimism.^47^ Whereas optimism predicts favorable outcomes, hope mobilizes effort toward meaningful goals regardless of outcome certainty. An optimist believes things will turn out well; a hopeful person searches for and pursues a way forward even when outcomes are uncertain or adverse.

Hope provides meaning, purpose, and emotional resilience. It does not eliminate suffering but situates the suffering within a broader narrative of value, connection, and coherence, rendering it more bearable.^48^^,^^49^

### Benefits of hope

Maintaining hope is associated with improved emotional and physical outcomes.^50^ Hope correlates with lower depression, greater life satisfaction, and enhanced psychological adjustment. It also encourages engagement in salutary behaviors, including adherence to medical regimens, rehabilitation efforts, and health-supportive routines.^41^^,^^51^

A hopeful patient may positively influence family members and caregivers, helping them manage emotional strain.^51^ Witnessing sustained hope can ease anticipatory grief, deepen shared presence, and facilitate meaningful connection. These shared processes may later support mourning and internalization of the patient, contributing to relatives’ own existential maturation and evolving engagement with mortality.

Hope serves as a foundation for emotional resilience, enabling patients to navigate the physical, psychological, social, and existential challenges of chronic and life-limiting illness while avoiding sustained despair.^44^^,^^52^ Even when curative treatment is no longer possible (or never was possible), hope permits anticipation of meaningful, attainable experiences: a visit with a loved one, participation in a valued role, immersing in a spiritual practice, or savoring a moment of peace amid symptoms.

Beyond immediate coping, hope may reflect existential maturation: the developing capacity to integrate awareness of mortality, limitation, and vulnerability in ways that support grounded engagement with life.^53^^,^^54^ Existential maturation is not inevitable; for some, confrontation with chronic decline intensifies fear and fragmentation, while for others, it deepens clarity regarding priorities and relationships.

### Why are some patients more hopeful?

Hopefulness varies widely across individuals.^55^^,^^56^ Objective measures of hope demonstrate variability in patients’ capacity to generate goals, identify pathways, and sustain agency.^55^ Individuals who have previously coped effectively with adversity often sustain hope more readily,^57^ focusing on what remains possible while integrating awareness of limitation.

The ability to recognize and value opportunities for connection, contribution, and meaning buffers despair and fosters hope. Even small domains of perceived control, such as daily routines, treatment decisions, and adaptive strategies, enhance agency. Engagement in meaningful activities, including valued relationships, legacy work, psychotherapy, peer support, spiritual practices, and sustained relational connection, reinforces purpose and sustains reasons to hope.^58^^,^^59^

### Contributors to hope

#### Physical factors

Effective symptom control is foundational for the preservation of hope. Relief from pain, breathlessness, fatigue, and other burdensome symptoms restores the capacity to engage with life.^1^^,^^60^^,^^61^ In chronic illness, even modest improvements in physical or cognitive functioning can meaningfully expand perceived possibility and agency. Conversely, poorly controlled symptoms constrain imagination of the future and undermine hope.

#### Relational factors

Human beings seek connection. Supportive relationships are among the strongest predictors of hope.^1^^,^^21^^,^^62^ Strong emotional bonds with family, friends, peers, and professional caregivers provide comfort, validation, and meaning. In chronic illness, sustained relationships over time can reinforce continuity of identity despite functional change.

Open, honest, and compassionate communication fosters trust and reinforces hope by affirming dignity and agency—even when discussing decline. Hope is not sustained by avoidance of reality but by relational authenticity within it.

#### Existential factors

For many patients, hope is not solely psychological but deeply spiritual in nature. Spirituality in this context should not be understood narrowly as religious belief or practice but more broadly as the search for meaning, connectedness, transcendence, coherence, reconciliation, and peace.^63^^,^^64^ Even patients who identify as secular may derive hope from existential frameworks that sustain continuity, purpose, and relational meaning in the face of suffering, uncertainty, and mortality.^64^

Patients derive hope from reflection on life’s meaning—relationships, contributions, moral commitments, spiritual beliefs, and narrative coherence. Spiritual or religious frameworks may offer continuity, transcendence, forgiveness, or reconciliation.^59^ Legacy-focused thinking—actions that preserve enduring presence in the lives of others—can bolster hope and provide psychological continuity beyond anticipated loss. In chronic illness, existential hope often shifts away from expectations of future achievement toward a process of integration: the effort to live in accordance with one’s values and sense of meaning despite enduring limitation.^65^

#### Cultural factors

Cultural frameworks shape how hope is conceptualized and expressed. Viewing death as a natural transition may support hope for peace and acceptance.^27^ In other contexts, hope may be sustained through communal solidarity, collective caregiving, prayer, ritual, or shared narrative meaning.^59^ Cultural norms also influence whether hope is expressed openly, privately, or symbolically.

Cultural frameworks may profoundly shape preferences regarding truth telling, prognostic disclosure, emotional expression, and decision-making authority. Clinicians should remain attentive to the risk of misinterpreting culturally shaped expressions of hope, silence, deference, or emotional restraint as maladaptive coping or denial.^29^ Consequently, approaches to communication are often shaped by how hope is understood, protected, and expressed within different families and cultures.

In some cultural contexts, family-centered or relational models of decision making may take precedence over individual autonomy-based approaches that predominate in Western medical ethics. Individual patients, however, may or may not subscribe to these norms.^66^ Culturally sensitive communication therefore requires exploration of individual preferences regarding information sharing, decision making, and maintenance of hope rather than culturally based assumptions.^66^

### Hope as an evolving adaptive process

Hope evolves across illness trajectories.^67^ In early phases, hope may center on cure or stabilization. In chronic disease, hope often recalibrates repeatedly toward functional maintenance, relational presence, or meaningful adaptation. As decline progresses, hope may orient toward achievable milestones, reconciliation, comfort, or spiritual growth. This evolution represents a transformation of hope, not its extinction. Near death, hope may focus on dignity, relational closure, or peaceful dying.^68^^,^^69^ The capacity to shift the focus of hope is a critical component of adaptive coping^70^ and a marker of existential maturation.^53^^,^^71^

#### Vignette 3: adaptive evolution of hope

A 52-year-old dentist with small-cell lung cancer initially pursued treatment in hope of cure. As his illness progressed and remissions shortened, his focus shifted to attending his daughter’s wedding and maintaining a part-time clinical involvement. Later, as functional decline accelerated, his hope centered on comfort, reconciliation, and dying at home. Each phase reflected adaptation rather than loss of hope; the object changed, but the orientation toward meaning endured.

### Maladaptive hope

Although hope is often adaptive, it may also assume maladaptive forms. Its value depends not simply on the presence of hope but also on its flexibility and capacity to coexist with evolving clinical realities. In advanced illness, excessively rigid, unrealistic, or externally reinforced hope for cure or remission may impair informed decision making, contribute to burdensome or nonbeneficial treatment, inhibit emotional processing, and delay practical or relational preparation for decline and death.^3^^,^^72^ This has been called the dark side of hope.^73^

Hope may function defensively as well as adaptively. Persistent insistence on cure or extraordinary recovery may at times reflect attempts to ward off overwhelming fear, despair, dependency, or existential distress.^74^ Clinicians may inadvertently reinforce these dynamics through prognostic avoidance or excessive emphasis on improbable benefit.^3^

The clinical task is to support the forms of hope that remain psychologically flexible, reality congruent, and capable of evolving over time. Adaptive hope allows mourning, preparation, meaning, and connection to coexist alongside uncertainty and possibility.

## Fostering adaptive hope in clinical practice

Addressing issues of hope is a central ethical and clinical responsibility in the care of patients, especially for those with chronic and life-limiting illness.^48^^,^^75^^,^^76^ Importantly, fostering hope does not require distortion of facts, avoidance of difficult conversations, or unrealistic reassurance.^1^^,^^77^ Rather, it entails a deliberate blending of honesty and compassion, realism and possibility—acknowledging what is lost while highlighting what remains achievable, meaningful, and life affirming.^1^^,^^78^

Approaches to support adaptive hope are fundamentally psychospiritual interventions that integrate communication, symptom management, relational presence, meaning-oriented reflection, and attention to existential suffering. Some interventions aim to generally foster hope, supporting adaptive coping, resilience, decision making, and emotional regulation. Others explicitly engage with hope as a therapeutic construct, helping patients articulate and strengthen personally significant goals.^71^

Professional, iterative support helps patients process complex emotions, confront existential concerns, and develop adaptive forms of hope that evolve across chronic and progressive illness trajectories. Practical implementation may include structured goals-of-care discussions, dignity-conserving interventions, meaning-centered psychotherapy, legacy work, spiritual care referral, reflective listening, and interdisciplinary team-based approaches.

Responsibility for nurturing hope is not confined to a single clinician: interdisciplinary collaboration—including psychologists, social workers, spiritual care providers, nurses, rehabilitation specialists, and palliative-care teams—aims to provide coordinated support addressing physical, emotional, relational, and spiritual domains.^79^ Optimal care requires coordinated communication and collaboration among oncology, palliative care, psycho-oncology, nursing, psychology, social work, rehabilitation, and spiritual care professionals whenever available.

This interdisciplinary collaboration is especially important in addressing maladaptive hope, demoralization, existential distress, caregiver burden, and complex communication surrounding uncertainty, deterioration, and end-of-life planning. Consistent, compassionate, and reality-congruent communication across disciplines helps patients and families maintain trust while supporting adaptive coping and meaning making.

### Empathic communication and holistic care

Empathic communication underpins all hope-fostering practice. It involves active listening, tolerating silence, and providing space for patients to express fear, anger, or ambivalence without premature reassurance. Through empathetic engagement, patients feel seen and understood, creating psychological safety within which hope can emerge.^1^^,^^80^^,^^81^

Hope-enhancing communication is inherently relational. Holistic, person-centered care reinforces hope by addressing physical, emotional, relational, cultural, and spiritual dimensions, signaling that the patient remains a whole person of inherent value.^1^^,^^76^^,^^82^ Respect for values, beliefs, and social context strengthens the environment in which adaptive hope can flourish, particularly in chronic illness where functional limitations and prolonged uncertainty challenge engagement and agency. In this way, communication and the therapeutic relationship affirm the patient’s dignity and agency, even in the context of chronic decline, functional limitation, or irreversible physical changes.

### Reframing and transitioning hopes

When cure or full recovery is not attainable, hope can be redirected toward attainable and meaningful domains: symptom control, functional maintenance, social engagement, personal goals, spiritual peace, reconciliation, and adaptation to new limitations.^68^^,^^83^, ^84^, ^85^ These alternative foci preserve engagement with life and the capacity for meaningful experiences.

Honest but compassionate communication is central to authentic hope. Patients can tolerate difficult truths when conveyed with empathy, clarity, and attentiveness to meaning and to the patient’s personhood.^1^^,^^77^^,^^86^ Avoidance of prognosis or euphemistic language may temporarily spare clinicians’ discomfort but often leaves patients isolated with unspoken fears, eroding trust and undermining hope for supportive accompaniment.^86^

Honesty regarding prognosis must not collapse into bluntness. A purely biomedical disclosure stripped of attention to meaning, goals, and personhood risks supplanting hope with despair. Clinicians’ task is to articulate medical reality while simultaneously allowing for mourning what has been lost and then reframing hope toward achievable and meaningful domains such as comfort, dignity, adaptation, connection, legacy, reconciliation, or spiritual peace^78^^,^^87^, ^88^, ^89^ (Table 1), while employing strategies that foster coping and adaptive engagement (Table 2). This reframing does not negate prior hopes but facilitates their evolution, shifting focus from external outcomes to relational, psychological, and existential domains.

**Table 1:** Integrated dimensions and examples of hope in life-limiting illness

Dimension of hope	Description	Illustrative examples of hope
Physical	Hope related to bodily comfort, symptom relief, and preservation of function	Hope for effective pain or symptom control; hope to maintain sufficient strength or mobility to participate in meaningful activities; hope for periods of physical stability
Cognitive/communication	Hope related to cognitive function and ability to communicate	Hope to remain clear headed and able to communicate effectively
Relational	Hope grounded in relationships, connection, reconciliation, and shared meaning	Hope to reconnect or mend strained relationships; hope to spend meaningful time with family and friends; hope to feel loved and not alone
Emotional	Hope that supports emotional well-being, resilience, and the capacity to experience connection and joy	Hope for moments of connection and joy or calm; hope for reduced fear or anxiety; hope to remain emotionally present with loved ones
Existential/spiritual	Hope related to meaning, purpose, faith, and transcendence beyond physical illness	Hope for peace of mind; hope for spiritual reconciliation or comfort; hope for transcendence or acceptance of suffering
Legacy oriented	Hope focused on what one leaves behind in values, memories, and impact	Hope to leave a legacy through letters, memories, or teachings; hope that one’s life has mattered
Family focused (future oriented)	Hope directed toward the future well-being of loved ones	Hope that a spouse will cope; hope that children will thrive; hope that family members will support one another

**Table 2:** Practical strategies to foster hope in clinical practice

Strategy	Core focus	Clinical application and rationale
Empathetic communication	Honest, compassionate dialog	Provide clear and truthful information about illness and prognosis while responding sensitively to emotional cues. Empathy validates suffering, builds trust, and creates psychological safety, allowing hope to emerge without denial of reality
Goal setting	Priorities and achievable aims	Set small, achievable goals: encourage patients to focus on realistic-term, meaningful goals
Talk therapy	Hopes, coping	Individual or group therapy
Facilitating social connection	Relationship-centered care	Encourage visits, reconciliation, shared conversations, and meaningful interactions with loved ones. Social connection counteracts isolation and reinforces relational sources of hope
Recognize achievements	Personal acknowledgement	Personal, professional, family, courage, resilience, faith, and honesty
Holistic care	Whole-person approach	Address physical, psychological, social, cultural, and spiritual needs in an integrated manner. When patients feel seen as whole persons rather than diagnoses, their sense of dignity and hope is reinforced
Encouraging meaning making	Purpose and coherence	Support patients in reflecting on their lives, values, relationships, and achievements. Meaning-making helps patients situate their illness within a broader life narrative, transforming despair into a sense of purpose. Talk therapy and group therapy can assist these processes
Respect for cultural beliefs	Cultural congruence	Tailor care to align with the patient’s cultural values, rituals, and beliefs about illness, death, and dying. Cultural attunement reduces alienation and strengthens trust, thereby sustaining hope
Reframing the narrative	Perspective	From loss to possibility, from control over outcomes to control over life priorities allows patients to experience hope without denying reality. This reframing does not minimize suffering but situates it within a broader context of meaning
Spiritual support	Existential and spiritual care	Offer access to chaplains, spiritual advisors, or counselors to address questions of faith, transcendence, guilt, forgiveness, and peace. Spiritual frameworks often provide enduring sources of hope beyond medical outcomes

Reframing can highlight possibilities previously eclipsed by the focus on disease and its management, encouraging engagement with relationships, unfinished tasks, and reflection on personal achievements or legacy. Activities such as creating family videos, journals, or other legacy projects can reinforce identity, purpose, and connection, further supporting hope.^1^^,^^90^, ^91^, ^92^ This capacity to reframe hope amidst illness is central to adaptive coping and a hallmark of existential maturation, enabling patients to confront uncertainty or mortality without becoming unmoored.^53^^,^^54^

Wish statements (e.g. ‘I wish I could help you get rid of this disease’) validate patients’ desires while aligning clinician and patient in shared acknowledgement of hope.^93^ Patients may sustain hopes for stabilization or functional improvement while simultaneously cultivating parallel hopes for quality of life, symptom relief, and meaningful experiences. Clinicians who master the skill of holding hope^88^—or supporting dual awareness (also termed dual coping^94^ or middle knowledge^95^)—enable patients to hope for the best while preparing for multiple possible outcomes.^96^ This is a nuanced and critical skill in chronic and life-limiting illness.

### The role of palliative and integrated medical care

Palliative care reinforces hope by addressing suffering across all domains—physical, emotional, relational, and spiritual.^1^^,^^48^^,^^75^^,^^76^ Symptom relief and functional support restore engagement and meaning, creating conditions conducive to hope.^97^ Beyond symptom management, palliative and rehabilitative care affirm inherent dignity, focusing on comfort, personhood, and individualized goals of care. Through ongoing presence, listening, and advocacy, care teams communicate that life remains valuable despite progressive limitation or uncertain prognosis.^98^ This approach has been described as intensive caring.^99^

### The role of family, caregivers, and community

Families, caregivers, and supportive social networks profoundly shape the patient’s hope environment.^100^^,^^101^ Honest dialog, emotional presence, and validation of realistic goals reinforce meaning, while avoidance of difficult conversations may inadvertently increase distress. Families may also facilitate legacy building, relational reconciliation, and small acts of encouragement, all potent sources of hope. Families are encouraged to recognize and validate the patient’s hopes without dismissing them or insisting on forced positivity.

At the same time, families themselves may struggle with anticipatory grief, caregiver burden, or uncertainty.^102^ Supporting caregivers emotionally is therefore compassionate and instrumental in preserving a hopeful environment for patients living with illness.

### Bolstering hope through spirituality and existential reflection

For many patients, hope is inseparable from spiritual or existential frameworks.^103^ These beliefs—religious or secular—provide coherence when medicine cannot promise a cure or functional restoration. Clinicians need not share these beliefs to support them but must be open and respectful.^104^ Indeed, spiritual concerns are often central rather than peripheral to the experience of serious illness. Patients often seek not only symptom relief or prolonged survival but also existential coherence, reconciliation, transcendence, and a sense that their lives retain meaning and value despite progressive illness.

Referral to spiritual care providers or faith leaders can support exploration of these issues. Spiritual frameworks often facilitate a transition of hope from survival or recovery to adaptation, transcendence, peace of mind, or trust.

Approaches used in spiritual and existential reflection may include religious or secular texts, prayer, life review,^105^^,^^106^ dignity therapy,^107^^,^^108^ narrative reconstruction,^109^ existential therapy,^110^ supportive-expressive group therapy,^111^ and meaning-making interventions.^39^^,^^112^^,^^113^ These approaches help patients articulate what has mattered most, what they are proud of, and how they wish to live despite limitations, transforming despair into a sense of coherence, purpose and, in some situations, closure.

### Hope augmentation tools

Structured interventions to enhance hope in clinical contexts have been developed, grounded in Snyder’s Hope Theory.^44^ These programs target the core components of hope: goal setting, pathway generation, and agency. Early high-dose, multisession approaches provided repeated exposure to hope-building strategies,^114^ while more recent low-dose or single-session formats offer pragmatic, clinically feasible alternatives.^115^^,^^116^

Most interventions employ hope mapping: identifying personally meaningful goals, anticipating barriers, and generating alternative pathways (Table 3). Some use collaborative, or dyadic, or group formats, reinforcing problem-solving skills and interpersonal support. These programs have evolved to include telephone, video, and digital applications, enhancing accessibility and scalability for patients living with chronic or progressive illness.^118^

**Table 3:** Hope augmentation techniques

Name	Design	Methods	Focus based on hope theory	Target audience	Outcomes	Notes	Reference
Manualized hope therapy	Multisession psychotherapy protocol	Goal clarification, barrier mapping, pathway generation, and agency self-talk training	Agency + pathways	Community adults	Pilot data showed increases in trait hope and well-being	Most structured model; closest to Snyder’s original theoretical architecture	^114^
Single-session goal pursuit intervention	90-minute session	Rapid goal identification, contingency planning, and motivational reframing	Primarily pathways, secondary agency	College students	Demonstrated measurable short-term hope change	Useful where session time is limited (e.g. oncology consult settings)	^116^
Metastatic breast cancer workshops	Facilitated group workshops	Values clarification, narrative reframing, collaborative goal setting, and clinician–patient alignment	Agency + meaning integration	Patients + oncologists	Pilot feasibility and acceptability confirmed	Notable for dual-audience design improving clinician communication behaviors	^115^
Pathways intervention	Structured multivisit clinical program	Structured goal navigation, coping rehearsal, and problem-solving scripts	Pathways-dominant	Patients with advanced lung cancer	Early trial suggests improved hope and reduced distress	Adaptable for integration into routine oncology visits	^117^
Online hope training for clinicians	Digital asynchronous modules	Reflective exercises, cognitive reframing, and scenario simulations	Agency + professional meaning	Oncology patients and professionals	Feasible and acceptable; improved self-reported hopefulness	Scalable and cost-efficient; supports workforce resilience	^118^
Third-wave integrated hope interventions	Conceptual synthesis + exemplars	Combines cognitive behavioural therapy, meaning-centered therapy, narrative medicine, and positive psychology	Multicomponent (agency + pathways + meaning)	Cross-population	Conceptual review identifying convergent model	Defines emerging paradigm: integrative, flexible, mechanism based	^113^
Meaning and purpose therapy	Manualized, individual psychotherapy; qualitative process analysis	Time-limited structured sessions exploring identity, meaning, legacy, relational narratives, and existential concerns	Strengthens goal reorientation, and agency facilitates adaptive shift of hopes to existential integration	Patients with advanced cancer experiencing existential distress	Reduced demoralization; enhanced coherence, meaning, and relational connectedness; adaptive hope	Emphasizes relational and narrative reconstruction; process focused	^119^^,^^120^

### Professional skill and resiliency development

The capacity to sustain adaptive hope in patients requires parallel development of clinicians’ own reflective, emotional, and existential capacities. Clinicians caring for patients with serious or life-limiting illness are repeatedly exposed to suffering, uncertainty, progressive decline, and death. Without opportunities for reflection and emotional processing, these experiences may contribute to avoidance behaviors, prognostic distancing, moral distress, compassion fatigue, and burnout.^121^ In such settings, clinicians may unconsciously resort to premature reassurance, excessive optimism, emotional withdrawal, or overtreatment as defenses against their own discomfort with helplessness, grief, or perceived failure.^3^^,^^122^

Educational approaches that cultivate self-awareness and reflective capacity are therefore essential components of serious illness communication training. These may include reflective writing, mindfulness-based practices, Balint-style discussion groups,^123^ facilitated debriefing,^124^ role-play with simulated patients,^124^ supervised clinical encounters, and experiential communication training focused on responding to emotion, uncertainty, and existential distress.^124^^,^^125^ Supporting clinicians in this way enhances not only clinician well-being but also the ability to foster flexible, reality congruent, and adaptive forms of hope in patients and families confronting serious illness.

## Hoping for miracles: Adaptive faith or defensive denial?

Hoping for miracles—or, in secular terms, hoping for an exceptional outcome is a common, psychologically intelligible response to life-limiting and chronic illness.^126^ Such hope can help patients contend with fear, uncertainty, and prolonged suffering, sustaining a sense of possibility even when conventional prognostic optimism is limited. It provides a form of agency that extends beyond the immediate scope of medical intervention and can serve as an emotional survival strategy, buffering overwhelming anxiety by allowing patients to imagine outcomes not constrained by present clinical realities.^72^^,^^127^

For many individuals, hope for exceptional outcomes is embedded within cultural, religious, or personal narratives. Trust in a higher power—or in the unknown—may provide comfort precisely at moments when medicine offers no guarantee of cure or functional restoration.^127^ Similarly, secular forms of miraculous hope, such as belief in exceptional recovery, late remission, or unforeseen breakthroughs, can reinforce engagement with life and facilitate emotional resilience, even amidst chronic or progressive illness.

### Clinical perspectives

Clinically, hope for miracles should neither be dismissed nor ridiculed.^126^ In many instances, it can coexist with realistic planning, adaptive goal setting, and advanced care preparation. Supporting a dual framework—acknowledging belief in extraordinary possibilities while simultaneously preparing for a range of probable outcomes—is both feasible and ethically appropriate. This approach respects the patient’s subjective experience of hope, while maintaining practical engagement with care decisions.

Hoping for a miraculous or exceptional outcome may also serve relational functions, strengthening bonds through shared rituals, communal narratives, or sustaining hope on behalf of loved ones.^128^^,^^129^ Spiritual care providers, chaplains, or culturally competent counselors can be invaluable in integrating faith or meaning-based commitments with medical understanding, helping patients reconcile extraordinary hope with adaptive decision making and realistic expectations.

### When hope for miracles becomes rigid

Concern arises when miraculous hope becomes inflexible: foreclosing discussion of prognosis, impairing informed decision making, or reflecting an inability to assimilate emerging realities. In such circumstances, hope may reflect defensive denial or demoralization rather than adaptive resilience.^74^

Clinicians must carefully discern whether miraculous hope coexists with realistic awareness or displaces it. When held alongside an accurate understanding, it can soften fear, maintain engagement, and preserve dignity; when rigid, it may erode trust, hinder adaptive coping, or delay practical planning.

#### Vignette 4: hoping for miracles with dual awareness

A deeply religious 35-year-old woman with a progressive, life-limiting illness spoke openly of praying for a miracle and trusting in divine healing. Simultaneously, she engaged in advance care planning, articulated her wishes for comfort-focused care, and spoke tenderly about saying goodbye to her family. Her hope for divine intervention did not preclude realism; rather, it enabled her to live meaningfully with uncertainty while preparing for all outcomes.

## Managing patients and family members who are unable to recalibrate hope

While many patients and family members demonstrate the capacity to recalibrate hope toward attainable and meaningful goals, some remain unable or unwilling.^130^ In such cases, hope may remain fixed on medically implausible goals or may collapse entirely into despair.^131^

### Failure to reframe hope

Not everyone has emotional flexibility. For some, the inability to reframe hope may be caused by overwhelming grief and fear, unresolved relational obligations, rigid cognitive styles, prior trauma, depression or demoralization, or the absence of viable alternative sources of meaning.

Clinical management begins with curiosity rather than correction. Clinicians should seek to understand what expressed hope represents. Questions such as ‘What feels most at stake if that does not happen’? or ‘What problem are we trying to solve’ may reveal underlying goals that may be more amenable to engagement. Patients or their family members do not need to relinquish the primary hope (e.g. for a cure or exceptional recovery) in order to develop additional hopes that are more immediately attainable. The clinician’s task is to gently expand the horizon of hope rather than replace it.

### Fixed rigid hope for cure or recovery

Situations when patients’ or family members’ hope remains rigid and exclusionary, especially when it leads to requests or demands for potentially harmful or inappropriate interventions, are more challenging. There is an ethically important distinction between respecting hope and colluding with harmful or inappropriate decision making.^132^^,^^133^ This requires an honest transparent communication regarding medical realities and professional responsibilities, framed with empathy and compassion. Clinicians should avoid harmful treatment or inappropriate resource utilization, while continuing to project empathy for the perspective of the patient or family members.^3^

### When hope is absent rather than rigid

When hope is absent rather than rigid, manifesting as pervasive despair and/or demoralization, the clinical priority shifts toward restoration of psychological and existential resources. This includes aggressive symptom control, treatment of depression and anxiety where present, and engagement in meaning-centered,^39^ dignity-conserving,^107^ or narrative-based interventions.^107^ Reframing hope in this context may initially be very modest, focusing on immediate and tangible experiences such as having less pain, presence, feeling the care of others, and genuine moments of human connection.

### Recognizing limits

Not all patients will achieve adaptive reframing of hope, and insistence that they do so may be counterproductive.^134^ In these situations, the inability to reframe hope should not be understood as failure but as a reflection of the profound difficulty of psychological and existential coping in the face of what is perceived to be catastrophic loss. Indeed, in some cases, maintaining a supportive relationship, minimizing harm, and accompanying the patient or a family member during their suffering may be the best we can do.^99^ In these instances, connection, commitment, and non-abandonment (intensive caring) are the essence of our therapeutic approach.

## Conclusion

Hope in the context of illness is neither synonymous with wishful thinking nor incompatible with realism. Rather, it is a dynamic and adaptive resource that enables people to live meaningfully even in the presence of chronic, progressive, or life-limiting disease. Although despair may arise as patients confront loss, uncertainty, and physical decline, it need not dominate the emotional landscape. Patients often sustain multiple forms of hope simultaneously—including hope for relief, for meaningful time with loved ones, for personal growth, or even for extraordinary outcomes. When hopes such as the hope for a miracle are approached respectfully and integrated with thoughtful clinical planning, they can coexist with acceptance, adaptive coping, and preparation for possible decline.

The findings of this review have important implications for clinical practice in oncology and palliative care (Table 4). Training programs in oncology, serious illness, and palliative care should include structured teaching in reflective communication, existential care, emotional self-awareness, and management of hope and despair in serious illness. Educational approaches incorporating reflective practice, experiential learning, supervision, and communication skills training may strengthen clinicians’ capacity to tolerate uncertainty and suffering while sustaining compassionate realism.

**Table 4:** Implications for practice

1.	Communication that supports adaptive hope should be recognized as a core clinical competency and intentionally integrated into routine care throughout the illness trajectory.
2.	Clinicians should be trained to foster adaptive hope through honest, compassionate communication that acknowledges suffering while helping patients identify meaningful and achievable goals.
3.	The concept of dual awareness, in which patients simultaneously sustain hope for the best possible outcome while preparing emotionally and practically for decline or death, should be normalized.
4.	Interdisciplinary models of care are particularly important because adaptive hope is shaped not only by medical information but also by psychological, relational, cultural, spiritual, and existential factors.
5.	Psychospiritual support provided by palliative-care clinicians, psychologists, social workers, spiritual care providers, rehabilitation specialists, and nurses should be fully integrated into comprehensive cancer care.

Further research is needed to evaluate hope-enhancing interventions across diverse populations and illness trajectories and to better distinguish adaptive from maladaptive forms of hope over time. Additional work should examine the cultural, spiritual, and relational determinants of hope, as well as the development and implementation of scalable models of hope-supportive care within oncology and serious illness practice.
